# Innovative Applications of Polymeric Foams

**DOI:** 10.3390/ma15228155

**Published:** 2022-11-17

**Authors:** Rezgar Hasanzadeh, Taher Azdast

**Affiliations:** Department of Mechanical Engineering, Urmia University, Urmia 5756151818, Iran

The new open Special Issue of *Materials*, entitled “Innovative Applications of Polymeric Foams”, aims to highlight original and review papers on new scientific and applied research and provide outstanding contributions to inform the community’s understanding of innovative applications of polymeric foams.

The applications of polymers are growing due to their special properties, such as low cost, light weight, and their high strength-to-weight ratio [1]. Achieving an efficient production method requires a decrease in the material consumption of polymers; additionally, product performance must be enhanced. Creating a cellular structure in polymers using the foaming procedure endows polymeric products with superior characteristics [2,3]. Foaming not only decreases the material consumption of polymers [4] but also improves different performances of the polymeric parts such as sound insulation [5], thermal insulation [6], piezoelectric performance [7], and electromagnetic interface shielding [8].

Saturating a polymer using a gaseous phase or by dispersing a chemical blowing agent is the first step in foaming a polymer; this is followed by a thermodynamic instability procedure for inducing cell nucleation. Thermodynamic instability is initiated by a temperature increment and/or a pressure drop [9]. Cell growth and cell stabilization are the other foaming steps. Cell density is controlled in the cell nucleation step; expansion is controlled in the cell growth stage [10].

Taking a retrospective view on research in this field that has been performed thus far, it seems amazing how fast polymeric foams have moved from scientific concepts to laboratory studies and to commercial products; they are due to form a vital part of life before the end of the present century. Currently, researchers have found that the mechanism of foam production is somewhat mysterious; the formation of a bubble—a combination of art and science—is fascinating in itself. Nowadays, polymeric foams have attracted particular attention in scientific and industrial societies due to their unique properties, such as a high strength-to-weight ratio, excellent thermal and sound insulation, desirable dielectric properties, innovative biomedical applications, and low cost. Statistically, polymeric foam production outputs 19.1 million tons of polymeric foam—with a worth of USD 87 billion—before 2019, which increased to 25.1 million tons in 2019, reaching an even higher amount in the past couple of years.

The research interests of the present Special Issue, “Innovative Applications of Polymeric Foams” include, but are not limited to, the following: the processing and manufacturing of polymeric foams; the morphological and structural properties of polymeric foams; the effect of processing parameters on the properties of polymeric foams; the relation of structural properties to the performance of polymeric foams; foam injection molding; foam extrusion molding; foam 3D printing; foam batch processing; innovative applications of polymeric foams based on their properties, such as thermal insulation, sound insulation, dielectric materials, piezoelectric materials, oil absorption, electromagnetic interface shielding, and biomedical applications.

## References

[1] Govaert L.E., van der Vegt A.K., van Drongelen M. (2020). Polymers: From Structure to Properties.

[2] Hasanzadeh R., Azdast T., Mojaver M., Darvishi M.M., Park C.B. (2022). Cost-effective and reproducible technologies for fabrication of tissue engineered scaffolds: The state-of-the-art and future perspectives. Polymer.

[3] Gama N.V., Ferreira A., Barros-Timmons A. (2018). Polyurethane foams: Past, present, and future. Materials.

[4] Azdast T., Hasanzadeh R., Lee R.E., Lee P.C., Wang G., Park C.B. (2022). High-Pressure Foam Injection Molding of Polylactide/Nano-Fibril Composites with Mold Opening. Polymeric Foams.

[5] Doyle L., Weidlich I., Di Maio E. (2022). Developing Insulating Polymeric Foams: Strategies and Research Needs from a Circular Economy Perspective. Materials.

[6] Hasanzadeh R., Azdast T., Doniavi A., Lee R.E. (2019). Multi-objective optimization of heat transfer mechanisms of microcellular polymeric foams from thermal-insulation point of view. Therm. Sci. Eng. Prog..

[7] Ansari M.A., Somdee P. (2022). Piezoelectric Polymeric Foams as Flexible Energy Harvesters: A Review. Adv. Energy Sustain. Res..

[8] Panahi-Sarmad M., Noroozi M., Xiao X., Park C.B. (2022). Recent Advances in Graphene-Based Polymer Nanocomposites and Foams for Electromagnetic Interference Shielding Applications. Ind. Eng. Chem. Res..

[9] Azdast T., Hasanzadeh R. (2021). Increasing cell density/decreasing cell size to produce microcellular and nanocellular thermoplastic foams: A review. J. Cell. Plast..

[10] Rostami M., Azdast T., Hasanzadeh R., Moradian M. (2021). A study on fabrication of nanocomposite polyethylene foam through extrusion foaming procedure. Cell. Polym..

